# Electroconvulsive therapy in patients with cranial metal implants

**DOI:** 10.1192/j.eurpsy.2025.2135

**Published:** 2025-08-26

**Authors:** P. Sulic, P. Viskovic, M. Zivkovic, A. Mihaljevic-Peles

**Affiliations:** 1 Department of Psychiatry, General Hospital “Dr. Ivo Pedišić” Sisak, Sisak; 2 The Child and Adolescent Psychiatry and Psychotherapy Unit, University Hospital Centre Zagreb; 3 Clinical Department of Psychiatry and Psychological Medicine, University of Zagreb School of Medicine; 4 Clinical Department of Psychiatry and Psychological Medicine, University Hospital Centre Zagreb, Zagreb, Croatia

## Abstract

**Introduction:**

Electroconvulsive therapy (ECT) is a widely recognized and effective treatment for severe psychiatric conditions, including major depressive disorder, treatment-resistant depression, and some psychotic disorders. Although ECT is highly effective, administering it to patients with cranial metal implants—such as plates, screws, clips, or electrodes—presents potential safety challenges. Metal implants, particularly those made of ferromagnetic materials, may interact with the electrical currents in ECT, which can lead to heating, shifting of the implant, or other adverse effects, thereby raising safety concerns.

**Objectives:**

To review the safety and clinical considerations of electroconvulsive therapy in patients with cranial metal implants. This literature search aims to identify evidence on risks, contraindications, and guidelines to support safe and effective treatment in this population.

**Methods:**

We conducted a literature search on PubMed to investigate the application of ECT in patients with cranial metal implants. The search included keywords “electroconvulsive therapy” and “ECT” combined with the terms “cranial metal implants,” “head metal implants,” and “metallic skull plate” interchangeably. We included only case reports. We used this approach to gather relevant studies addressing the safety, efficacy, and considerations of electroconvulsive therapy in individuals with metal implants in or near the skull.

**Results:**

The literature search identified 11 case reports documenting the successful administration of ECT in patients with cranial metal implants. Across these cases, ECT was performed without adverse events related to the implants, and treatment outcomes were reported as effective in managing psychiatric symptoms. Most reports emphasized careful planning and individualized assessment to minimize risks, particularly regarding implant location and material composition. Safety measures included conducting pre-treatment imaging to evaluate implant positioning.

**Conclusions:**

The connection between cranial metal implants and ECT necessitates careful consideration of safety protocols to prevent potentially hazardous interactions. While metals such as titanium are generally not problematic for this therapy, it is essential to consider the type of metal, its location, and its magnetic properties before applying this technique. Our clinical experience also shows positive outcomes in patients with cranial metal implants, demonstrating safe and effective administration with proper evaluation and precautions. Further research and establishing safety guidelines will be crucial for optimizing treatment and minimizing risks for patients with metal implants.

**Disclosure of Interest:**

None Declared

